# MicroRNAs Dysregulation and Metabolism in Multiple System Atrophy

**DOI:** 10.3389/fnins.2019.01103

**Published:** 2019-10-17

**Authors:** Chunchen Xiang, Shunchang Han, Jianfei Nao, Shuyan Cong

**Affiliations:** Department of Neurology, Shengjing Hospital of China Medical University, Shenyang, China

**Keywords:** microRNA, multiple system atrophy, alpha-synuclein, autophagy, neuroinflammation

## Abstract

Multiple system atrophy (MSA) is an adult onset, fatal disease, characterized by an accumulation of alpha-synuclein (α-syn) in oligodendroglial cells. MicroRNAs (miRNAs) are small non-coding RNAs involved in post-translational regulation and several biological processes. Disruption of miRNA-related pathways in the central nervous system (CNS) plays an important role in the pathogenesis of neurodegenerative diseases, including MSA. While the exact mechanisms underlying miRNAs in the pathogenesis of MSA remain unclear, it is known that miRNAs can repress the translation of messenger RNAs (mRNAs) that regulate the following pathogenesis associated with MSA: autophagy, neuroinflammation, α-syn accumulation, synaptic transmission, oxidative stress, and apoptosis. In this review, the metabolism of miRNAs and their functional roles in the pathogenesis of MSA are discussed, thereby highlighting miRNAs as potential new biomarkers for the diagnosis of MSA and in increasing our understanding of the disease process.

## Introduction

Multiple system atrophy (MSA) is a progressive, fatal neurodegenerative disease. Two types of MSA are clinically distinguished: the parkinsonian variant (MSA-P), associated with striatonigral degeneration, and the cerebellar variant (MSA-C), related to olivopontocerebellar atrophy (Gilman et al., 2008; Stefanova et al., 2009; Jellinger, 2014). In Western countries, MSA-P is the most common variant of MSA (Gilman et al., 2008). In contrast, a recent study in Chinese patients found no significant difference between the number of MSA-P and MSA-C patients (Zhang et al., 2018). MSA is primarily a sporadic disease; familial MSA has also been reported (Multiple-System Atrophy Research Collaboration, 2013). Currently, there are no effective therapies for MSA treatment, only symptomatic therapy (Fanciulli and Wenning, 2015).

Multiple system atrophy is characterized by the accumulation of misfolded alpha-synuclein (α-syn) in oligodendroglial cells. Abnormal α-syn is also the pathological feature of other neurodegenerative diseases including Parkinson’s disease (PD) (Nuber et al., 2018; Palma and Kaufmann, 2018). While abnormal α-syn is evident in both MSA and PD cases, when brain extracts of α-syn from MSA and PD cases were injected into transgenic mice, only the α-syn from MSA cases induced neurodegeneration. These results indicate that MSA strains of α-syn are more toxic than the PD strains of α-syn in terms of neurodegeneration (Prusiner et al., 2015).

MicroRNAs (miRNAs) are small non-coding RNAs (19–24 nucleotides in length) that regulate messenger RNA (mRNA) expression and control post-translational regulation (Treiber et al., 2019). Interestingly, it is possible that miRNAs may modulate MSA-related gene expression, for example, SNCA encoding α-syn (Asi et al., 2014; Tagliafierro and Chiba-Falek, 2016). However, MSA-related genes, such as small nuclear ribonucleoprotein polypeptide N (SNRPN), may, in turn, regulate miRNA processing (Xiong et al., 2015; Hama et al., 2017). It is well known that miRNAs in serum, plasma, and cerebrospinal fluid (CSF) are tissue-specific, highly stable, and quantifiable, indicating that these miRNAs may be used to provide early and a more accurate diagnosis of several diseases, including neurodegenerative diseases (Ramaswamy et al., 2018). Targeting miRNAs by anti-miRs have shown positive results in several preclinical studies in cancer and various other diseases (Rupaimoole and Slack, 2017). The role of antisense oligonucleotides (ASO), structurally similar to anti-miRs, is also being investigated in preclinical and clinical trials in several neurodegenerative diseases including PD, amyotrophic lateral sclerosis (ALS), Huntington disease (HD), and Alzheimer disease (AD) (Kordasiewicz et al., 2012; Hinrich et al., 2016; Becker et al., 2017; Zhao et al., 2017). The therapeutic potential of anti-miRs and miR mimics in neurodegenerative diseases has been demonstrated (Zhou et al., 2016; Valera et al., 2017).

The pathophysiological mechanisms of MSA remain unknown; however, studies have demonstrated that α-syn toxicity contributes to the disruption of multiple organelles, including mitochondria, synaptic vesicles, lysosomes, and autophagosomes, and the nucleus, all of which are involved in the pathogenesis of MSA (Abati et al., 2018). Furthermore, α-syn toxicity has been shown to contribute to neuroinflammation, loss of neurotrophic support and neuronal dysfunction, resulting ultimately in neuronal death (Wong and Krainc, 2017). It is possible that miRNAs may regulate targeted genes and play a role in the pathogenesis of MSA. In this review, the role of MSA-related genes and the regulatory network between miRNAs and mRNAs will be discussed in the hope of providing new insight into the early diagnosis and therapeutic treatment of MSA.

## Biology of miRNAs

It is known that miRNAs control the expression of more than 50% of protein-coding genes by acting as post-translational regulators (Krol et al., 2010). Disruption of miRNAs can cause mitochondrial dysfunction, oxidative stress, and cell death (Arshad et al., 2017; Treiber et al., 2019). More specifically, miRNAs also play a role in the proliferation of neural stem cells, the maturation of neurons, and the formation of synapses (Bian and Sun, 2011).

**FIGURE 1:**
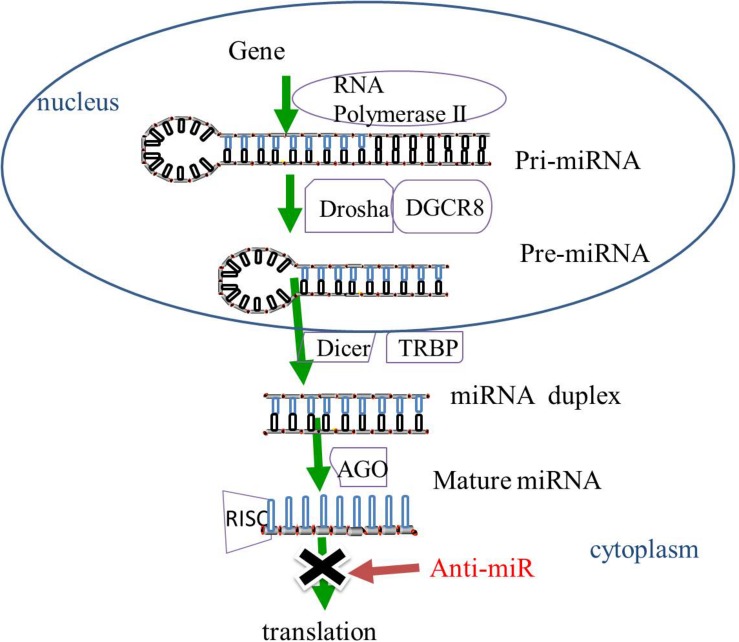
The biogenesis of miRNAs. In the nucleus, miRNAs are transcribed by RNA polymerase II into pri-miRNAs, and cleaved by RNase III Drosha and DGCR8 to pre-miRNAs. The pre-miRNAs are released from the nucleus to the cytoplasm and sliced by RNase III protein Dicer and TRBP to form miRNA duplexes. The miRNA duplexes are incorporated into the RISC, which is mediated by the AGO family, leading to the inhibition of gene expression. MiRNA-based treatment, including anti-miR can rescue the repression of miRNA-targeted genes. Pri-miRNA, primary miRNA; Pre-miRNA RNase, ribonuclease; DGCR8, cofactor DiGeorge Critical Region 8; TRBP, *trans-*activation-responsive RNA binding protein; RISC, RNA-induced silencing complex, AGO, Argonaute; anti-miR, anti-microRNA.

Gene-encoding miRNAs are transcribed by RNA polymerase II into primary miRNAs (pri-miRNAs). The pri-miRNAs then undergo cleavage by ribonuclease (RNase) III Drosha and cofactor protein DiGeorge Critical Region 8 (DGCR8) to form pre-miRNAs that are released from the nucleus to the cytoplasm. The pre-miRNAs in the cytoplasm are then sliced by RNase III protein Dicer and *trans-*activation-responsive RNA binding protein (TRBP) to form miRNA duplexes. The miRNA duplexes are incorporated into the RNA-induced silencing complex (RISC) mediated by the Argonaute (AGO) family. After unwinding and strand selection, the mature one-strand miRNA is capable of target recognition. The mature miRNAs then guide RISC to the complementary sequences for the 3′-untranslated region of the target mRNAs, resulting in the repression of mRNA-induced genes (Bartel, 2009; Huntzinger and Izaurralde, 2011; Figure 1).

Mutation of the genes encoding the miRNAs biogenesis enzymes, Drosha, Dicer, and AGO has been reported to be linked to several types of cancer and neurodegenerative diseases (Tan et al., 2015). The absence of Dicer has been shown to cause a reduction in the expression of dopamine neurons and a stimulation of miRNAs, thereby promoting neuronal survival, resulting in implications for the treatment of PD (Chmielarz et al., 2017).

The *trans-*activating response region (TAR) DNA-binding protein-43 (TDP-43) facilitates the production of pre-miRNAs via interaction with the Drosha complex and has been shown to be important in the pathogenesis of ALS (Kawahara and Mieda-Sato, 2012). The colocalization of TDP-43 and α-syn in glial cytoplasmic inclusions (GCIs) has been reported in a small number of MSA patients (Geser et al., 2011; Koga et al., 2018).

Induction of mRNA degradation and inhibition of mRNA translation by miRNAs highlight the important roles of miRNAs in several biological processes. In addition, neurodegenerative disease-related protein has also been reported to affect miRNA expression (Kawahara and Mieda-Sato, 2012; Rinchetti et al., 2018).

## The Role of miRNAs in MSA Pathogenesis

**FIGURE 2:**
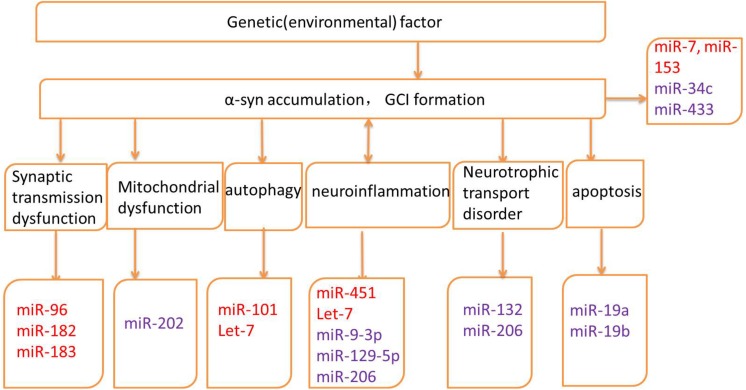
Pathogenic mechanisms and related microRNAs (miRNAs) of MSA. Red words indicate the up-regulated miRNAs and purple words indicate the down-regulated miRNAs.

Several genes may be related to the pathogenesis of MSA; however, the exact mechanisms and the roles of these genes remain unknown (Mitsui et al., 2015; Sailer et al., 2016; Gu et al., 2018; Table 1). Altered expression of transcripts related to myelination and neuroinflammation has been observed in MSA striatum (Kim et al., 2019). In addition, a progressive decay of genes related to glutamate transport was reported (Kim et al., 2019). Therefore, miRNAs may regulate translation in neuronal processes and activation in synaptic transmission, thus taking part in various biological processes in neurodegenerative diseases, including MSA. The potential mechanisms underlying miRNA dysregulation will now be discussed, and the different roles of miRNAs in MSA pathogenesis will be further described in the hope of highlighting the development of potential biomarkers and therapeutic approaches (Figure 2 and Table 2).

**TABLE 1:** Summary of MSA-related genes and their function.

**Genes**	**Functions**	**Results**	**References**
SNCA	α-Synuclein gene, encoding α-synuclein protein	Variants rs3857059, rs3822086, and rs3775444 are the risk factors for MSA, while rs2736990, rs11931074, and rs356220 are irrelevant	Al-Chalabi et al., 2009; Scholz et al., 2009; Guo et al., 2014; Sailer et al., 2016
COQ2	Involved in the biosynthetic pathway for coenzyme Q10	Variant rs397514727 is associated with sporadic MSA while other results failed to find the association	Multiple-System Atrophy Research Collaboration, 2013; Sailer et al., 2016
MAPT	Microtubule-associated protein tau gene	Controversial: H1 haplotype is harmful, while H2 haplotype is protective. SNP rs9303521 increases the risk for MSA	Vilarino-Guell et al., 2011; Labbe et al., 2016; Sailer et al., 2016; Gu et al., 2018
IL-1β	Inflammatory-related genes	Variant rs16944 of IL-1β might be the gene factors that modified the age at onset in MSA, variant rs1799964 of TNF-α increases risk for MSA	Zhou et al., 2018
TNF-α			
GBA	Glucocerebrosidase gene, the pathogenic genes for Gaucher disease	Variant rs76763715 of GBA is associated with MSA-C patients	Mitsui et al., 2015
FBXO47	F-box protein 47, promoting ubiquitination	Controversial: Variant rs78523330 for FBXO47 might be associated with MSA, and others failed to find similar results	Sailer et al., 2016; Gu et al., 2018
EDN1	Endothelin 1, maintain vascular tone	Controversial: Variant rs16872704 for EDN1 might be associated with MSA, and others failed to find similar results	
SHC2	Src homology 2 domain containing-transforming protein 2, signaling adapter	Copy number loss of SHC2 strongly indicates a causal link to MSA	Sasaki et al., 2011; Ferguson et al., 2014
SLC1A4	Solute carrier family 1A4	Variant rs759458 and haplotype “T-C-C-G” and “T-C-T-A” for SLC1A4 associated with MSA-C patients	Soma et al., 2008
SNRPN	Small nuclear ribonucleoprotein polypeptide N, encoding protein required for miRNA biogenesis	Homozygous deletions of SNRPN are risk factors for MSA patients	Hama et al., 2017
NMD3	Nonsense-mediated decay 3, regulating mRNA and rRNA nuclear export	Variant rs34016896 has an increased risk for MSA in female patients	Chen et al., 2018

**TABLE 2:** Summary of altered microRNAs (miRNAs) and their targeted genes in Multiple system atrophy (MSA).

**MicroRNAs**	**References**	**Resources**	**Regulation**	**Target genes**	**Roles**
MiR-7, miR-153	Doxakis, 2010; Ubhi et al., 2014	α-syn primary neuron/human brain and mouse	Increase/No alteration	*SNCA*	Glial cytoplasmic inclusions component
MiR-34c	Marques et al., 2017; Valera et al., 2017	Cerebrospinal fluid/striatum	Decrease		
MiR-433	Lee et al., 2015; Schafferer et al., 2016	Human brain, mouse	Decrease	*HDAC6*	
MiR-132	Lee et al., 2015; Wakabayashi et al., 2016	Human brain, formalin-fixed paraffin-embedded	Decrease	*BDNF*	Neurotrophic support
MiR-206	Lee et al., 2015	Human brain	Decrease	*IGF-1*	
				*NR4A2*	Neuroinflammation
MiR-451	Kume et al., 2018	Serum	Increase	*TLR4*	
MiR-129-5p	Lee et al., 2015; Wakabayashi et al., 2016	Human brain, formalin-fixed paraffin-embedded	Decrease	*TLR3*	
MiR-9-3p	Starhof et al., 2018a	Cerebrospinal fluid	Decrease	*NLRP3*	
Let-7	Valera et al., 2017; Starhof et al., 2018a	Cerebrospinal fluid	Increase	*TLR7*	
				*MTOR*	Autophagy
MiR-101	Valera et al., 2017	Cerebrospinal fluid	Increase	*ATG4D*	
MiR-202	Lee et al., 2015	Human brain	Decrease	*POU2F1*	Oxidative stress
MiR-96, miR-182, miR-183	Ubhi et al., 2014	Human brain, mice	Increase	*SLC1A1*, *SLC6A6*	Synaptic transport
miR-19a, miR-19b	Marques et al., 2017	Cerebrospinal fluid	Decrease	*CASP9, TP53*	Apoptosis

### miRNAs and GCI Aggregation

The aggregation of α-syn promotes the relocalization of tubulin polymerization-promoting protein (TPPP/p25α) from myelin to oligodendroglia, resulting in oligodendrocyte swelling and abnormal uptake of α-syn by oligodendrocytes (Asi et al., 2014; Reyes et al., 2014). The interaction between α-syn and p25α also promotes the phosphorylation of α-syn and the formation of insoluble GCIs (Jellinger and Wenning, 2016). The formation of GCIs disrupts neuronal support, neuroinflammation, and neurotrophic support (Fanciulli and Wenning, 2015). It has been shown that miRNAs play important roles in the pathogenesis of MSA by regulating the expression of GCI components, α-syn, heat-shock protein (HSP), and p25α (Wong and Krainc, 2017). Both miR-7 and miR-153 have a similar expression and distribution pattern with α-syn in both neural and non-neural tissues. In addition, it has been shown that overexpression of miR-7 and miR-153 significantly reduces the level of α-syn in primary neurons (Doxakis, 2010). A study in MSA patients was unable to replicate these findings (Ubhi et al., 2014). Interestingly, it has been reported that miR-34c is reduced in the striatum of MSA-P patients (Valera et al., 2017) and in the CSF of MSA patients (Marques et al., 2017). Indeed, inhibition of miR-34c has been shown to result in the aggregation of α-syn in a cellular model (Kabaria et al., 2015).

Along with hyperphosphorylated α-syn, GCIs also contain ubiquitin, HSP, and p25α (Jellinger and Lantos, 2010). It has been suggested that down-regulation of miR-433 in the striatum observed in an MSA transgenic mouse model may be associated with the regulation of histone deacetylase 6 (HDAC6), a microtubule-associated deacetylase (Schafferer et al., 2016). These findings were further supported by a study showing colocalization of GCIs immunolabeled with anti-HDAC6 antibody in the striatum from MSA patients (Chiba et al., 2012). To conclude, miRNAs are possibly involved in the pathogenesis of MSA via the regulation of GCI components. It is therefore possible that miR-7, miR-153, and miR-34c may act as neuroprotective agents by regulating the SNCA; however, the precise roles of these miRs in MSA require further investigation.

### miRNAs and Neuroinflammation

Neuroinflammation is a dynamic response including activation of microglia and astroglia, the expression of proinflammatory cytokines and chemokines (O’Callaghan et al., 2008). The role of neuroinflammation in the pathogenesis of MSA has been demonstrated in several studies (Vieira et al., 2015). Recent studies have suggested that microglial activation is essential to α-syn accumulation and promotes cell degeneration in neurodegenerative diseases (Sanchez-Guajardo et al., 2015). Indeed pro-inflammatory cytokines are elevated in serum, CSF, and brain tissue of MSA patients (Kaufman et al., 2013; Rydbirk et al., 2017; Starhof et al., 2018b).

Toll-like receptor (TLR) mediates the activation of innate immunity (Takeda and Akira, 2005). Furthermore, TLR4 mediates α-syn-induced microglial activation and pro-inflammatory cytokines. The mRNA expression of several TLRs is elevated in MSA, including TLR-3 and TLR-4 (Fellner et al., 2013).

Up-regulation of miRNAs can repress neuroinflammation and act as a positive feedback, reducing cell death in the pathogenesis of MSA. Overexpression of miR-451 has been shown to inhibit the release of cytokines through microglial activation, via the targeting of TLR4 (Sun and Zhang, 2018). Levels of miR-451 are elevated in brains of MSA patients (Kume et al., 2018).

Other miRNAs have also been shown to promote neuroinflammation and induce neurodegeneration. Up-regulation of Let-7 in MSA is associated with the activation of microglia and the induction of neurodegeneration by activating TLR7 (Lehmann et al., 2012). Elevated miR-129-5p can decrease cytokine activation and ameliorate inflammation-induced neuronal damage via TLR3 (Li et al., 2017). In addition, expression of miR-129-5p is decreased in MSA patients (Lee et al., 2015; Wakabayashi et al., 2016). In human astrocytes, overexpression of miR-206 increases pro-inflammatory cytokine expression (Duan et al., 2015), and miR-206 is down-regulated in the brains of MSA patients (Lee et al., 2015).

The nucleotide-binding domain leucine-rich repeats protein family (NLRP3) inflammasome is a multiprotein cytosolic complex that induces the release of cytokines (Duewell et al., 2010). The number of NLRP3 inflammasome-related proteins is increased in the brains of MSA patients postmortem. In a study by Li et al., NLRP3 inflammasome-related proteins played a role in astroglial activation and the release of interleukin-1 beta (IL-1β) (Li et al., 2018). It has also been shown that miR-9 can decrease NLRP3 expression, resulting in the suppression of pro-inflammatory cytokines (Wang et al., 2017). Levels of miR-9-3p are down-regulated in the CSF of MSA patients compared to PD patients, indicating that this miR may be used as a biomarker to discriminate between MSA and PD (Starhof et al., 2018a).

Release of α-syn by degenerating neurons may induce neuroinflammation, and simultaneously, neuroinflammation may trigger cytokine release, thus producing a pro-inflammatory environment, leading to the formation of intracellular α-syn aggregates (Vieira et al., 2015). Furthermore, the miRNA-based therapeutic strategies have been reported to be successful via regulating neuroinflammation in several neurodegenerative diseases, further highlighting a role of miRNAs in neuroinflammation in the treatment of MSA (Gaudet et al., 2018).

### miRNAs and Autophagy

Disruption in the clearance of aggregated proteins is well known in the pathogenesis of neurodegenerative diseases (Scrivo et al., 2018). Selective autophagy may therefore act as a potential target for some neurodegenerative diseases. The inhibition of autophagy has been related to elevated secretion and transmission of α-syn (Lee et al., 2013). Autophagy is primarily controlled by autophagy-related proteins (ATG) and the mammalian/mechanistic target of rapamycin (mTOR) family (Sarkar, 2013). It has been shown that the mTOR pathway is involved in the pathogenesis of MSA and that mRNA levels of mTOR are decreased in the striatum of MSA patients (Valera et al., 2017). The conjugation of ATGs with microtubule-associated protein 1 light chain 3 (LC3) forms autophagosome, a double-membrane vesicle. Immunoreactivity of LC3 has been associated with α-syn-positive GCIs in neuropathological examination of MSA brains (Schwarz et al., 2012). The phosphorylation regulated by kinase complex beclin-1 has also been shown to be important in the formation of autophagosomes (Scrivo et al., 2018). Furthermore, expression of beclin-1 is decreased in MSA patients (Kaji et al., 2018; Miki et al., 2018).

It has been shown that miRNAs are associated with the pathogenesis of MSA by specifically targeting autophagy, including miR-101 and Let-7. In association with the increase of miR-101, levels of the autophagy markers beclin-1 and LC3 were decreased in the striatum of MSA-P patients. In an MSA mouse model, treatment with anti-miR-101 increased levels of LC3 and beclin-1, resulting in improved autophagy (Valera et al., 2017). Furthermore, treatment with the Let-7 family has been shown to inhibit the mTOR signaling pathway, in conjunction with a significant increase in the levels of Let-7b in the brains of MSA patients (Dubinsky et al., 2014; Valera et al., 2017).

Autophagy is involved in the process of neuroinflammation in neurodegenerative diseases (Su et al., 2016). Enhancing autophagy, as indicated by the up-regulation of beclin-1 and autophagy-related 5 (ATG5), facilitates the shift from deleterious microglial response M1 to neuroprotective microglial response M2 (Ji et al., 2018). Inhibition of the mTOR pathway has been shown to reduce neuronal death and microglial activation (Srivastava et al., 2016).

To conclude, levels of miR-101 and Let-7b are up-regulated through the ATG and the mTOR family in the pathogenesis of MSA. Results of a cellular model demonstrated the suppression of autophagy through regulation of miRNAs, indicating a role for anti-miR-101 in the treatment of MSA (Valera et al., 2017). The miRNAs, such as Let-7, show potential in the regulation of the pathogenesis of MSA via different biological processes.

### miRNAs and Deregulation of Neurotrophic Factors

Neurotrophic factors, including glial-derived neurotrophic factor (GDNF), brain-derived neurotrophic factor (BDNF), and insulin-like growth factor (IGF) can mediate the development, survival, and maintenance of the peripheral system and the CNS (Bianchi et al., 2017; Sampaio et al., 2017). Neurotrophic factors are produced by oligodendrocytes (Ubhi et al., 2010) and the deregulation of these factors play important roles in the pathogenesis of MSA. Serum IGF-1 is significantly higher in MSA patients and is associated with disease progression (Numao et al., 2014). It has been reported that BDNF and other neurotrophic factors are elevated in MSA patients, while a specific reduction in the expression of GDNF was observed in α-syn transgenic mice and in MSA patients (Ubhi et al., 2010; Rydbirk et al., 2017). In conjunction, these findings suggest that increased expression of IGF-1 and BDNF may act as a compensatory response in MSA.

The expression of miR-132 was down-regulated in the brain of postmortem MSA patients (Lee et al., 2015; Wakabayashi et al., 2016). Up-regulation of miR-132 decreased the expression of BDNF, thereby inhibiting neuronal survival (Lungu et al., 2013). The relationship between the down-regulation of miR-206 in MSA patients and neuroinflammation has been previously reported (Lee et al., 2015). An additional study has shown that miR-206 is negatively associated with the IGF-1 signaling pathway, suggesting that IGF-1 may be another target gene for miR-206 in the pathogenesis of MSA (Xing et al., 2016; Liu et al., 2018).

### miRNAs and Oxidative Stress

Oxidative stress can promote α-syn accumulation in oligodendrocytes (Pukass et al., 2015). Progressive microglial activation can cause chronic oxidative stress and ultimately lead to neuronal cell death in an MSA transgenic mouse model (Stefanova et al., 2007). The organic cation transporter proteins (OCT) are transcription factors that regulate gene levels by regulating reactive oxygen species (ROS). OCT is widely expressed by Purkinje cells in the cerebellum. Low levels of OCT1 in the MSA cerebellum has been shown to result in reduced resistance of neurons to oxidative stress (Lee et al., 2015). Up-regulation of miR-202 has been shown to down-regulate the expression of OCT1, thus contributing to cerebellar degeneration in the cerebellum of MSA patients (Lee et al., 2015).

### miRNAs and Synaptic Transmission Dysfunction

Elevated α-syn expression has been shown to be increased in the presynaptic terminal, thus disrupting dopamine and γ-aminobutyric acid (GABA) release (Guatteo et al., 2017). Neurotransmitters at synapses are transported to presynaptic terminals through several transporters, including SLC1 and SLC6 (Lin et al., 2015). The SLC1A1 and SLC6A6 genes, which encode for the neuronal/epithelial high-affinity glutamate transporter (EAAT3/EAAC1) and taurine transporter, respectively, are down-regulated by the miR-96 cluster (miR-96, miR-182, miR-183) in MSA brain tissues and MSA mouse models, indicating a role for miRNAs in neurotransmitter release (Ubhi et al., 2014).

### miRNAs and Apoptosis

The accumulation of apoptosome-related protein can occur in the neuronal and oligodendroglial elements of the MSA brain. Activated caspase-9 and -8, and increased expression of p53, have been observed in MSA brain tissue (Kawamoto et al., 2016). A reduction of miR-19a and miR-19b has been reported to result in a significant increase in apoptosis in SH-SY5Y cells and increase the level of p53 protein in MSA brain tissue (Zhu et al., 2016). In addition, levels of both miR-19a and miR-19b have been found to be lower in the CSF of MSA patients in the early stage of the disease (Marques et al., 2017). A recent study also found that miR-19b was decreased in the plasma of MSA patients compared to controls and PD patients. Interestingly, levels of miR-24 were also decreased in the plasma of MSA patients, with a strong correlation between miR-24 and miR-19b levels, suggesting that both miR-24 and miR-19b play a role in the regulation of apoptosis and the pathogenesis of MSA (Uwatoko et al., 2019).

## miRNAs as Biomarkers of Msa

Several parameters, including α-syn, total tau, phosphorylated tau, the 42-amino-acid form of Aβ, neurofilament light chain protein, fms-like tyrosine kinase ligand, homocysteine, uric acid, and coenzyme Q10, have previously been identified as potential biomarkers for the diagnosis of patients with MSA (Laurens et al., 2015). However, these results are inconsistent and several biomarkers are unable to differentiate among neurodegenerative diseases characterized by the abnormal accumulation of α-syn.

Recently, miRNAs have been recognized as potential biomarkers for MSA due to their small size, stability, accuracy, and tissue-specific nature (Mitchell et al., 2008). Several studies have shown that miR-9-3p, miR-19a, miR-19b, and miR-24 are potential biomarkers that can be used to distinguish patients with MSA from those with PD and healthy people (Marques et al., 2017; Starhof et al., 2018a; Uwatoko et al., 2019). However, the expression of miRNAs is differentially expressed in the CSF and peripheral blood of patients with MSA (Vallelunga et al., 2014; Marques et al., 2017). The inconsistency of results may be due to the small sample sizes of the studies. Further clinical investigations with a large sample size, along with functional studies, are needed to confirm the role of miRNAs in the pathogenesis of MSA.

Interestingly, miRNAs can also be used as prognostic factors for nervous system diseases. For example, similar to the results reported using samples from the CSF of patients with MSA, miR-19a and miR-19b were also down-regulated in the prodromal stage of patients with synucleinopathies (Fernández-Santiago et al., 2015). In contrast, the up-regulation of both miR-19a and miR-19b has been reported in MSA patients at postmortem (Ubhi et al., 2014). Together, these findings show that the expression of miRNAs is not consistent throughout the course of the disease, indicating that miRNAs may be useful in determining disease stage.

## miRNA-Based Therapeutic Approaches for MSA

Based on current studies, blocking α-syn aggregation (rifampicin), enhancing neuroprotection (riluzole), and reducing neuroinflammation (minocycline) were expected to have positive effects on the treatment of MSA (Bensimon et al., 2009; Dodel et al., 2010; Low et al., 2014). Unfortunately, most of these treatments failed in clinical trials and more effective therapies are therefore urgently needed.

RNA interference-based clinical trials by miRNA mimics for various diseases are progressing well (Bobbin and Rossi, 2016). Reducing gene expression by miRNA has also been shown to be effective in neurodegenerative diseases (Keiser et al., 2016). For example, anti–miR-101 improved autophagy and reduced α-syn accumulation in an MSA mouse model (Valera et al., 2017). MiR-7 reduced α-syn accumulation and had a neuroprotective effect in both cellular and mouse models, which demonstrate that miR-7 may be helpful in the treatment of diseases characterized by α-syn accumulation (Junn et al., 2009; Titze-de-Almeida and Titze-de-Almeida, 2018). However, an additional challenge exists for miRNA mimics in the treatment of neurodegenerative diseases due to the blood–brain barrier. Virus vector-mediated delivery and specifically designed polymeric nanoparticles may be useful; however, further studies are needed (Saraiva et al., 2016; Nakamori et al., 2019).

## Conclusion

Several studies have shown that miRNAs have the potential to be considered as biomarkers and prognostic factors in MSA. MiR-9-3p, miR-19a, miR-19b, and miR-24 may be regarded as potential biomarkers to distinguish between MSA and PD patients, and healthy people, while the expression of miR-19a and miR-19b may be related to the development of the disease. Furthermore, miRNA mimics and anti-miR could reduce α-syn expression and play a beneficial role in both cellular and mouse models. Thus, the development of miRNA-based treatment may provide a new therapeutic model for disease modification in MSA. In addition, the role of miRNAs in MSA remains to be further elucidated by large-scale comparative studies.

## Author Contributions

CX and SC contributed to the drafting and revising the manuscript. SH contributed to drafting and modifying the table. JN contributed to drafting and modifying the figure. All authors approved the final version of the manuscript and agreed to be accountable for all aspects of the work in ensuring that questions related to the accuracy or integrity of any part of the work are appropriately investigated and resolved.

## Conflict of Interest

The authors declare that the research was conducted in the absence of any commercial or financial relationships that could be construed as a potential conflict of interest.
